# Molecular mechanisms of the anti-cancer drug, LY2874455, in overcoming the FGFR4 mutation-based resistance

**DOI:** 10.1038/s41598-021-96159-0

**Published:** 2021-08-16

**Authors:** Fariba Dehghanian, Shahryar Alavi

**Affiliations:** Department of Cell and Molecular Biology & Microbiology, Faculty of Biological Science and Technology, University of Isfahan, Isfahan, Iran

**Keywords:** Cancer, Computational biology and bioinformatics, Genetics, Molecular biology

## Abstract

In recent years, many strategies have been used to overcome the fibroblast growth factor receptor (FGFR) tyrosine kinase inhibitors (TKIs) resistance caused by different mutations. LY2874455 (or 6LF) is a pan-FGFR inhibitor which is identified as the most efficient TKI for all resistant mutations in FGFRs. Here, we perform a comparative dynamics study of wild type (WT) and the FGFR4 V550L mutant for better understanding of the 6LF inhibition mechanism. Our results confirm that the pan-FGFR inhibitor 6LF can bind efficiently to both WT and V550L FGFR4. Moreover, the communication network analysis indicates that in apo-WT FGFR4, αD–αE loop behaves like a switch between open and close states of the substrate-binding pocket in searching of its ligand. In contrast, V550L mutation induces the active conformation of the FGFR4 substrate-binding pocket through disruption of αD–αE loop and αG helix anti-correlation. Interestingly, 6LF binding causes the rigidity of hinge and αD helix regions, which results in overcoming V550L induced resistance. Collectively, the results of this study would be informative for designing more efficient TKIs for more effective targeting of the FGFR signaling pathway.

## Introduction

Fibroblast growth factor receptors (FGFRs) are a family of transmembrane tyrosine kinases which are involved in various biological processes through activation of the phosphoinositide-3-kinase (PI3K)/AKT and mitogen-activated protein kinase (MAPK) pathways^1^. FGFRs have been reported as risk factors in different human diseases, including cancers^2^, diabetes^3^, lung^4^, and heart diseases^5^. Although FGFR4 is identified as the last discovered FGFR, its dysregulation in cancers has been reported in many studies in recent years. It has been shown that FGFR4 gene mutation, overexpression, and amplification increase the incidence and development of different types of cancers including cancers of breast, liver, colon, prostate, and rhabdomyosarcoma and hepatocellular carcinoma^6–9^.

FGFR4 contains three domains, including an extracellular ligand-binding domain (residues 1–369), a transmembrane helix (residues 370–390), and an intracellular tyrosine kinase domain (residues 391–802). FGFR4 kinase domain structure consists of two lobes, namely N (L467–E551) and C (N557–V755) lobes, which are bridged by a disordered hinge region (C552–G556). In physiological conditions, the binding of fibroblast growth factors (FGFs) to the extracellular domain induces receptor dimerization, resulting in autophosphorylation and/or phosphorylation of kinase domains^10–12^. The activity of the FGFR4 cytoplasmic domain is regulated through the phosphorylation of some of its tyrosine residues. The un-phosphorylated FGFR4 is inactive mainly due to the gate-keeper residues (Asp-Phe-Gly or DFG motif) regulating the hinge region of the kinase domain. DFG motif is located on A-loop, and its conformation is identified as an indicator of kinase activity. A hydrogen bonding network between N535, E551, and K627, a salt bridge between K503 and E520, and a hydrogen bond between R650 and D612 residues keep the inactive close state of the substrate-binding pocket of the FGFR4 kinase domain^13^. Some FGFR4 gate-keeper mutations, such as N535K and V550E, could cause sustained activation of FGFR4, which consequently leads to proliferation, survival, and metastasis of cancer cells^14,15^.

Receptor tyrosine kinase inhibitors (TKIs) such as ponatinib and dovitinib are used as therapeutic factors for FGFR-related cancers. However, patients with gate-keeper mutations, such as V550L, have intrinsic resistance to these therapies. For example, ponatinib sterically clashes with the extra methyl group of the mutant L550 residue^13^. To overcome the drug resistance of the FGFR4 V550L mutant, a novel drug candidate, the LY2874455 (or 6LF), has been used in clinical trials. 6LF is a type I pan-FGFR inhibitor; it binds to the ATP-binding pocket of the receptor and does not induce any conformational changes. 6LF can overcome the V550L mutation-based resistance as it is far from the gate-keeper residues when bound to the substrate-binding pocket^10,16,17^. The molecular mechanisms by which the 6LF inhibits the V550L mutant have not yet been fully addressed. Molecular dynamics (MD) simulations are noteworthy for drug discovery investigations^18^. Therefore, in the present study, we aim to conduct a pharmacodynamics study of 6LF to determine the effectiveness and the molecular mechanism of this candidate drug in inhibition of the FGFR4 V550L mutant. Our results indicate that the apo-WT FGFR4 switches between open and close states to search for 6LF. The V550L mutation induces active conformation of the enzyme’s substrate binding pocket, and 6LF binding results in the rigidity of regulatory regions, which in turn inhibits the enzyme activity.

## Results

**Figure 1:**
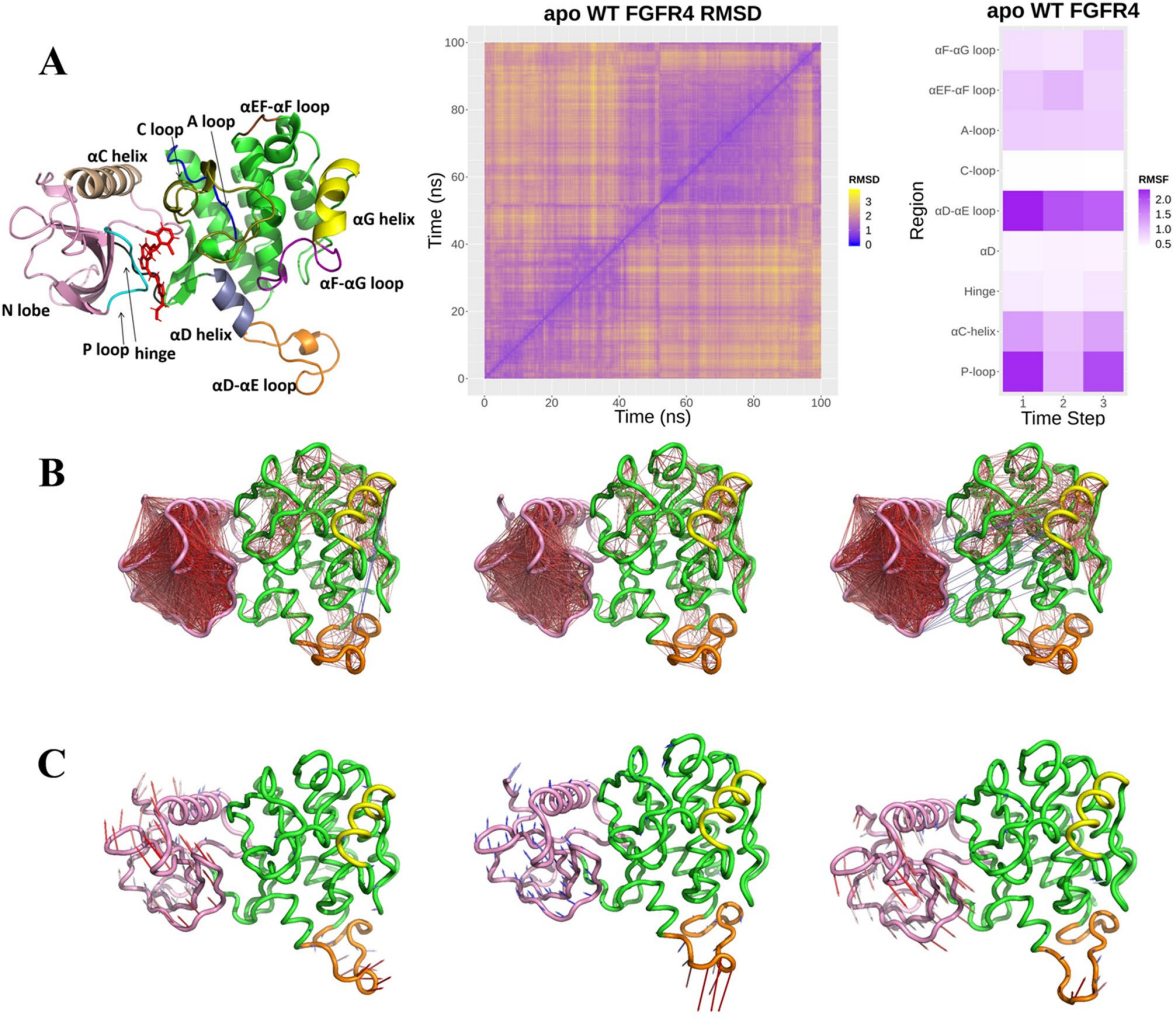
The apo-WT FGFR4 is in search of its substrate. (**A**) 6LF-WT FGFR4 structure, representing the protein moiety we used as the input structure for the simulation systems. 6LF is in red sticks. FGFR4 protein regions are colored as: N-lobe in pink, αG helix in yellow, αD–αE loop in orange, αF–αG loop in purple, αEF–αF loop in brown, A loop in olive, C loop in blue, P loop in cyan, αC helix in wheat, hinge in black, αD helix in gray, and other residues in green. (**B**) pairwise RMSD heat map. Based on this, the apo-WT-FGFR4 simulation was divided into three steps: 0–50, 50–90, and 90–100 ns. (**C**) Time stepped RMSF heat map. This indicates the most fluctuations in the αD–αE loop, αG helix, and P-loop regions. (**D**) Dynamics cross-correlation representation. Residues with highly correlated or anti-correlated motions are linked by a red or blue line, respectively. (**E**) PC1 motion. Arrows indicate the direction and contribution of the residues in the protein’s significant conformational motion.

All systems were run for 100 ns, but, the pairwise RMSD results indicated that the 100 ns of the simulation was not enough (Supplementary Figure 1). Therfore, all simulations were continued for another 100 ns to overcome highly fluctuation during the first 100 ns caused by missing residue in reference structure. The results of pairwise RMSD analysis for the second 100 ns indicated a relatively stable conformation of all systems (Supplementary Figure 2), and thus we did all of our analysis (below) on the second 100 ns simulations. Based on pairwise RMSD analysis, the second 100 ns of simulations were divided into different steps (Fig. 1B,C). The significant step of each simulation, which contains the most stable conformation during 100 ns, was selected for comparison of four systems. All steps of each simulation and the selected one for comparison have been indicated in Supplementary Table 1. The structure Coordinates of FGFR4 for further analysis were also represented in Table 1.

**Table 1:** The structure coordinates of FGFR4.

Structure coordinates		
	Residue numbers (UniProt)	Residue numbers (in simulated system)
N lobe	454–551	1–97 (pink region in structures)
P-loop	475–479	21–25
β2–β3 loop	488–496	34–42
β3–αC loop	503–509	49–55
αC	510–528	56–74
β4	536–540	82–86
Hinge	552–556	98–102
αD	557–564	103–111
αD–αE loop	565–584	112–131 (orange region in structures)
αE loop	585–606	131–152
C-loop	607–613	152–159
β6–β7 loop	621–625	167–171
A-loop	629–650	175–196
αEF	651–664	197–210
αEF–αF loop	665–666	211–212
αF	667–684	213–230
αF–αG loop	685–694	231–240
αG	694–704	241–251 (yellow region in structures)
αG–αH loop	705–714	252–260
αH	715–726	261–272
αH–αI loop	727–734	273–280
αI	735–750	281–296

### The apo-WT FGFR4 is in search of its substrate

In apo-WT FGFR4, the β2–β3 loop has highly fluctuated during the 100 ns, but it does not have a major contribution in PC1 motions. Its dynamics are anti-correlated with P-loop and β3–αC loop (Fig. 1D). High fluctuation of P-loop and β3–αC loop as the major contributions in PC1 motion (Fig. 1E) during the 0–50 ns suggests that the β2–β3 loop is pushing the P-loop and β3–αC loop. During 50–90 ns, the anti-correlation of β2–β3 with P-loop and β3–αC loop has been disappeared, and these regions no longer contribute to PC1 motions, and their fluctuations have been reduced. The αD–αE loop was anti-correlated with αG, which in turn is correlated with αD–αE. αD–αE loop has a significant contribution in PC1 motion, and αG has a minor contribution. During 50–90 ns, fluctuations of αG have been reduced, and its anti-correlation with an αD–αE loop has been disappeared, leading to an increase in αD–αE loop contribution in PC1 motions. This suggests that αG inhibits motions of the αD–αE loop. During 90–100 ns, PC1 contributions are very similar to that of 0–50 ns, but the motions are in a reverse direction. N-lobe DCCM shows that the anti-correlation of the β2–β3 loop has been disappeared, but the N-lobe dynamics has a new anti-correlation with αG. αG fluctuations, instead of inhibiting motions of the αD–αE loop, inducing dynamics of N-lobe in the opposite direction compared to 0–50 ns. Also, the αD–αE loop is correlated with αG–αH loop. It seems that in the apo WT system, the enzyme is in search of its substrate and the αD–αE loop behaves like a switch between open and close states.

Altogether, when αD–αE loop switches αG off, resulting in the formation of an N-lobe correlation network, in which β2–β3 loop induces motions of P-loop and β3–αC loop, leading to coordinated motion of N-lobe towards the open state. These motions continue until the inducing force is reduced and correlations are weakened. Now αD–αE loop switches αG on, resulting in the formation of a new correlation network in which αG dynamics, instead of a β2–β3 loop, induces motions of N-lobe, and consequently forces back the N-lobe to close state.

### V550L mutation induces active conformation of the enzyme’s substrate-binding pocket

**Figure 2:**
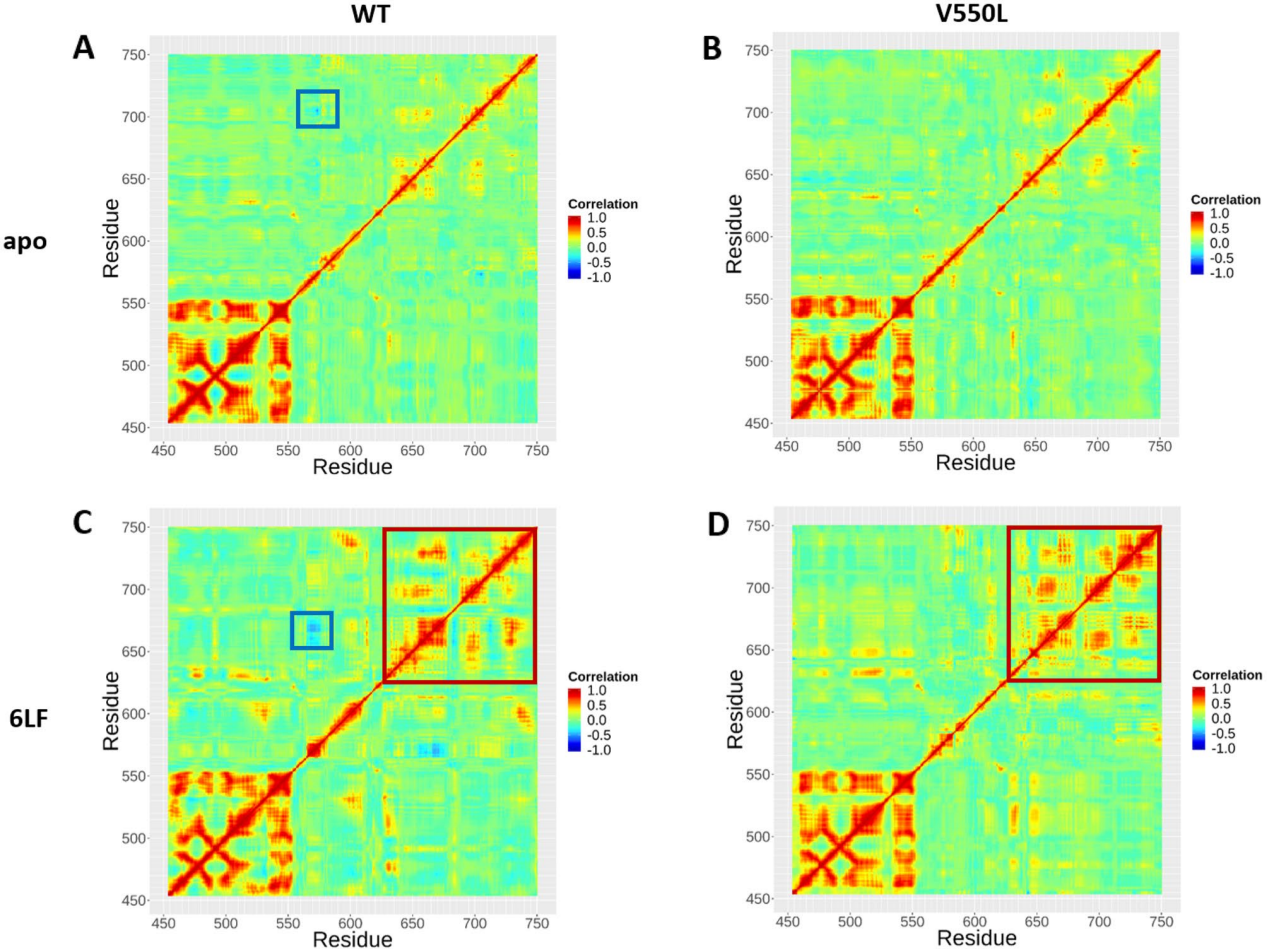
Dynamics cross-correlation map (DCCM) of the four FGFR4 simulations. Each dot in these maps shows a pairwise correlation of motions of FGFR4 residues. Highly positively correlated or negatively correlated motions are depicted by red or blue dots, respectively. Significant differences among the four systems are boxed (and further discussed in the text).

**Figure 3:**
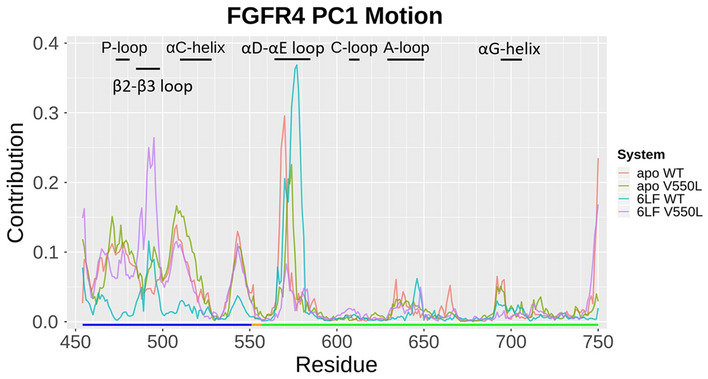
Residues’ contribution in the significant conformational motions. For each system, the 1st principal component motion was selected, and then the contribution of each residue in that motion was calculated.

V550L mutation results in disruption of anti-correlation of the αD–αE loop with αG helix (Fig. 2). Analysis of the apo-WT system shows that this correlation has a major role in regulating the enzyme’s substrate-binding pocket conformation. In WT systems (both apo and 6LF-liganded), αD–αE loop motions allosterically affect αG dynamics, which indicates the conformational state of the substrate-binding pocket. Nevertheless, V550L mutation adjusts this correlation network, induces contribution of the β2–β3 loop in PC1 motions (Fig. 3). According to the main role of β2–β3 loop in P-loop motions regulation and subsequently substrate binding, our results suggests that V550L mutation induces active conformation of the enzyme’s substrate-binding pocket throughout the activation of β2–β3 loop dynamics. Interaction of 6LF with P-loop reduces β2–β3 loop fluctuations, which may decrease enzyme activity. As β2–β3 loop controls P-loop motions, which involves substrate binding, it suggests that V550L mutation induces active conformation of the enzyme’s substrate-binding pocket throughout the activation of β2–β3 loop dynamics. As 6LF interacts with P-loop, it reduces β2–β3 loop fluctuations, which may decrease enzyme activity.

### 6LF binding results in the rigidity of hinge and αD helix regions

**Figure 4:**
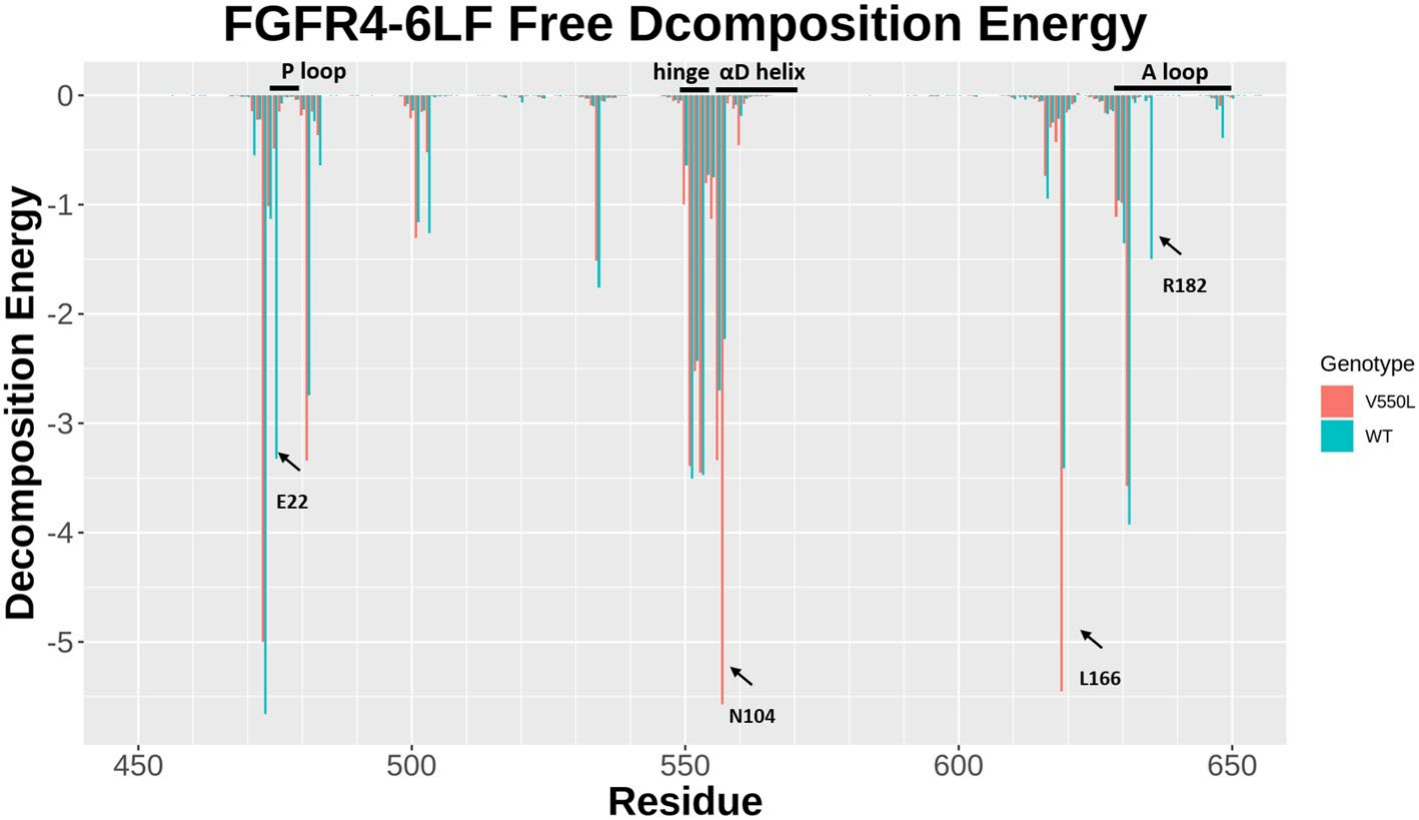
6LF binding energy. Major FGFR4 residues that participate in 6LF binding are different between the WT and the V550L mutant. However, the total energy (kcal/mol) released upon ligand decomposition is approximately equal between the two proteins.

The relative binding affinity between 6LF and either the wild-type or V550L-mutant FGFR4 was calculated using the MMPBSA method. The details of binding free energies are shown in Table 2. By adding the entropy values (− TΔS), the total value of the binding Gibbs free energy was calculated for each complex. The final Gibbs binding free energies for 6LF-WT and 6LF-V550L FGFR4 complexes were found to be − 26.9841 kcal/mol and − 25.3474 kcal/mol, respectively. Our result supports the idea that 6LF can efficiently bind to both WT and V550L FGFR4 complexes. In order to determine a detailed pattern of the binding energy in WT and V550L FGFR4 complexes, the binding free energy was decomposed into 6LF-residue pairs (Fig. 4). Our analysis relating to the effect of V550L mutation on the pattern of binding energy shows that the binding affinity of 6LF in the mutant complex is approximately the same in the WT complex. The most important residues in 6LF binding in both WT and V550L complexes are highlighted in yellow in Supplementary Table 2. Residues of P-loop, hinge, αD helix, and A loop are involved in binding free energy of both WT and mutant complexes. The most important differences in binding patterns are related to E22, N104, L166, and R182 (all are highlighted in blue in Supplementary Table 2). E22 from P-loop has significantly more contribution in WT compared to mutant complex. Furthermore, the N104 from αD helix shows more contribution in the V550L mutant complex. The R182 is only involved in the binding of the WT, while L166 is only contributed to V550L mutant complex binding energy. Therefore, the comparison of apo systems with 6LF-liganded systems shows that 6LF binding, causes the rigidity of hinge and αD helix regions. Free decomposition energy analysis shows that this region, in both WT and V550L, strongly interacts with 6LF. Also, P-loop, A-loop, hinge, and αD helix regions participate in the stabilization of 6LF in both WT and V550L complexes.

**Table 2:** Free energy results from MMPBSA.

Complex	ΔE^ele a^	ΔE^vdw b^	ΔG^PB c^	ΔG^SA d^	ΔG^MMPBSA^	TΔS	ΔG^binding^
6LF-WT FGFR4	− 24.5739 (0.1213)	− 48.6688 (0.0794)	35.4890 (0.0914)	− 6.1838 (0.0076)	− 43.9375 (0.1042)	− 16.9534 (1.3747)	− 26.9841 (1.9465)
6LF-V550L FGFR4	− 22.7990 (0.1004)	− 46.9144 (0.0716)	32.6805 (0.0796)	− 5.7547 (0.0065)	− 42.7750 (0.1000)	− 17.4403 (1.3747)	− 25.3474 (1.3747)

Standard errors of corresponding values are given in parentheses. All units are given in kcal/mol.

^a^ΔE^ele^: Electrostatic energy.

^b^ΔE^vdw^: Van der Waals energy.

^c^ΔG^PB^: Poisson–Boltzmann polar solvation energy.

^d^ΔG^SA^: Non-polar solvation energy.

## Discussion

The family of fibroblast growth factor receptors (FGFRs) comprises four highly preserved tyrosine kinase receptors (TKRs), including FGFR1, FGFR2, FGFR3, and FGFR4. All 23 FGF ligands exert their functions through these four TKRs^19^. FGF/FGFR-mediated signaling pathways play oncogenic roles in different types of cancers as they induce tumor angiogenesis, tumor cell survival, growth, and migration. The inactivation and blocking of FGF/FGFR signaling using TKIs is an approved therapeutic strategy in tumor-targeted therapy^20,21^. However, there is an essential concern about resistance to the FGFR inhibitors resulting from FGFRs gate-keeper mutations. The FGFR inhibitors side effects, such as hypertension and bleeding, resulted from their activity against vascular endothelial growth factor receptor 2 (VEGFR2) is another concern which should be considered^2,22^. Interestingly, the 6LF was reported as the most effective TKIs against the FGFR gate-keeper mutations, lacking the critical activity against VEGFR2^17^. The clinical phase I study also reported the excellent activity and tolerability of 6LF in patients with advanced cancer^16,23^. Recently, FGFR4 has received a great deal of attention from researchers due to its roles in tumorigenesis and anti-tumor therapy resistance in different cancers. Dysregulation of FGFR4 had been reported in different types of cancers through different molecular mechanisms, including the FGFR4 and its ligand overexpression, FGFR4 single nucleotide polymorphisms (SNPs), as well as somatic mutations. Like other TKRs, FGFR4 contains three main regions including extracellular, transmembrane and intracellular tyrosine kinase domains^24,25^. The intracellular kinase domain contains a N-terminal domain (N-lobe) and a C-terminal domain (C-lobe). There is a cleft between these two lobes which contains the FGFR4 active site. Three disordered regulatory regions including activation loop (A-loop, A629–R650), phosphate binding loop (P-loop, L473–V481) and catalytic loop (C-loop, K607–L613) are located in these lobes. The αC-helix (S510–G528) as a regulatory helix and a beta barrel like structure make the N-lobe. The FGFR4 phosphorylation sites and other α helices are located in C-lobe. DFG-motif in A-loop is identified as the indicator of FGFR4 kinase activity. ATP is surrounded by P-loop and His-Arg-Asp residues from C-loop are involved in catalyzing the transferring of the ATP phosphate group to the substrate^10,24^.

It has been shown that some mutations in N535 and V550 kinase domain residues of the FGFR4 located result in the more stable activity of the FGF/FGFR4 pathway in different types of cancers. The mutations in V550 residues of the FGFR4, which are identified as gate-keeper mutations, prevented the TKIs from efficient binding to FGFR4. V550 residue is localized in the Hinge region, which plays a role in regulating the active conformation of FGFR4 and the access of TKIs to the ATP binding pocket. Recently, Wu et al*.* have proved that Pan-FGFR inhibitor 6LF has a significant activity to overcome the resistance resulted from FGFR4 V550L mutation. Furthermore, they performed a crystallographic experiment indicated that 6LF is far from the gate-keeper residue^10,17^.

In this study, MD simulations were used to investigate the molecular mechanism by which 6LF inhibits the FGFR4 V550L mutant. MD simulation is a robust tool for understanding biological mechanisms at the atomic level^26^. To the best of our knowledge, this is the first study that investigates the molecular dynamics of pan-FGFR inhibitor 6LF activity against V550L mutation to find out FGFR4 structure-to-function relationships. Overall, our MD simulations have clearly confirmed that: (1) V550L mutation induces active conformation of the enzyme’s substrate-binding pocket, (2) 6LF binding results in the rigidity of hinge and αD helix regions, and P-loop, A-loop, hinge, and αD helix regions participate in the stabilization of 6LF in WT and V550L mutant complexes, and (3) 6LF can efficiently bind to both WT and V550L FGFR4 complexes.

Wu et al*.* reported two specific conformational states, including DFG-in and DFG-out for FGFR4 in complex with different TKIs^10^. The DFG-in was considered an FGFR4 active and open conformation, and the DFG-out was proposed as FGFR4 inactive and closed conformation. In agreement with their results, our MD simulations indicate that apo-WT FGFR4 is shifting between the two open and close states during 100 ns simulation. Further network correlation analyses suggest a regulatory network for switching between these two states. We suggest the involvement of αD–αE loop, β2–β3 loop, P-loop, and αG helix in the regulation of this correlation network.

Moreover, V550L mutation results in disruption of anti-correlation of the αD–αE loop with αG helix, which plays a key role in regulating the conformation of enzyme’s substrate-binding pocket. Therefore, V550L mutation induces active conformation of the enzyme’s substrate-binding pocket throughout the activation of β2–β3 loop dynamics. 6LF binding to P-loop results in the reduction of β2–β3 loop fluctuations, rigidity of hinge and αD helix regions, which in turn could decrease the FGFR4 enzyme activity.

## Conclusion

Here, we proved the ability of 6LF in dominating the FGFR4 V550L drug resistance. Also, we proposed the molecular mechanism to elaborate how 6LF inhibits the kinase activity of the mutant V550L FGFR4. 6LF has the potential to be a targeted treatment option for personalized medicine in cancer patients who suffer from FGFR4 V550L drug resistance.

## Materials and methods

### Structure preparation

Four systems including apo-WT FGFR4 (PDB ID: 4QQT), apo-V550L FGFR4 (PDB ID: 4QQJ), 6LF-liganded FGFR4 (PDB ID: 5JKG), and 6LF-liganded V550L FGFR4 (PDB ID: 5XFF) were designed (Fig. 1A). FGFR4 residues 454–750 were selected in all systems, and missing residues were taken from PDB ID 4QRC (Table 3). Three missing coiled regions of these systems were added by superimposing the coiled regions from PBD ID 4QRC.

**Table 3:** FGFR4 systems properties.

System	Structure reference (PDP ID)	Residue numbers (UniProt)	Residue numbers (in simulated system)
apo-WT FGFR4	4QQT	454–750	1–296
apo-V550L FGFR4	4QQJ	454–750	1–296
6LF-liganded WT FGFR4	5JKG	454–750	1–296
6LF-liganded V550L FGFR4	5XFF	454–750	1–296

### Molecular dynamics simulations

The four FGFR4 systems were further prepared for MD simulation using Amber18. The Amber ff14SB and GAFF (Generalized AMBER Force Field) force fields were used for protein and ligands, respectively. The ligands AM1-BCC partial atomic charges were assigned using the Antechamber modules^27^. The Leap module of Amber was utilized to add missing hydrogens, neutralize systems, provide the physiologic salt concentration, and generate the parameters and coordinate files for MD simulations. All complexes were solvated in an octahedral TIP3P (20) water model box with at least 15 Angstrom distance between protein complex and water box edges with Na and Cl ions to neutralize the system and keep the 0.15 M physiologic salt concentration. The systems were gone through 4000 cycles of minimization, with restraints on the FGR4 (and 6LF in the liganded systems) in the first 1500 cycles. Then, the systems were heated up slowly from 0 to 310 K in 500 ps with restraints on the FGFR4 (and 6LF in the liganded systems), followed by equilibration in 1 ns. Finally, all systems were subjected to production MD simulation, using NPT conditions and non-bonded cut-off distance as 12 Angstroms. Each system was run for 200 ns using a GPU-accelerated version of Amber18^28–30^. For hydrogen bonds, the SHAKE algorithm was utilized, and 2 femtoseconds time step was considered. Coordinates were captured every 10 ps.

### Trajectory analysis

Analyses were performed using CPPTRAJ^31^ and R package Bio3D 2.4^32^. The overall conformational changes of all trajectories were confirmed by evaluating the pairwise root-mean-square deviation (RMSD) of the backbone using the Bio3D package. Moreover, the root mean square fluctuation (RMSF) of each residue compared with the average structure of that residue was analyzed throughout the trajectory. RMSF analysis was considered to determine regions responsible for significant conformational changes. Furthermore, the dynamic cross-correlation (DCC) analysis was used to study the fluctuations and movements in the backbone of the Cα atoms. DCC is a statistical analysis determining how much two residues are similar in their movement during a selected simulation time. Principal component (PC) analysis was done to retrieve the main motions of the protein residues. For PC and DCC analyses, the most rigid region of each system was detected, and then all of the trajectories were superimposed based on this region. The molecular visualizations were created using PyMol 2.4, and plots were created using the ggplot2 package.

### MMPBSA and normal mode analyses

The differences between protein and 6LF binding energies in WT and V550L mutant complexes were calculated using molecular mechanics Poisson–Boltzmann surface area (MMPBSA) method^33^. The free energy calculations were done for 100 snapshots extracted from 100 ns MD trajectory. Normal-mode analysis was performed using the nabnmode module^34^ of Amber to estimate the conformational entropy contributions to the binding free energy. Finally, the free energy decomposition analysis was performed to investigate the interaction energy profiles of 6LF and FGFR4. The pairwise decomposition was analyzed in order to calculate the interaction energy between 6LF and all residues in the system by the MMGBSA decomposition process in the MMPBSA module of Amber18. This analysis results in descriptions of the binding mode of 6LF in WT and V550L mutant FGFR4 complexes.

## Supplementary Information

Supplementary Information 1.

## Data Availability

Data sharing not applicable to this article as no datasets were generated during the current study.
