# Supplementary material for: Molecular mechanisms of the anti-cancer drug, LY2874455, in overcoming the FGFR4 mutation-based resistance
Source: Sci Rep. 2021 Aug 16;11:16593. doi: 10.1038/s41598-021-96159-0 (PMC8368202; doi:10.1038/s41598-021-96159-0)
Supplement: Supplementary file 1 — Supplementary Information 1. [file 41598_2021_96159_MOESM1_ESM.docx]

**Supplementary Figures**


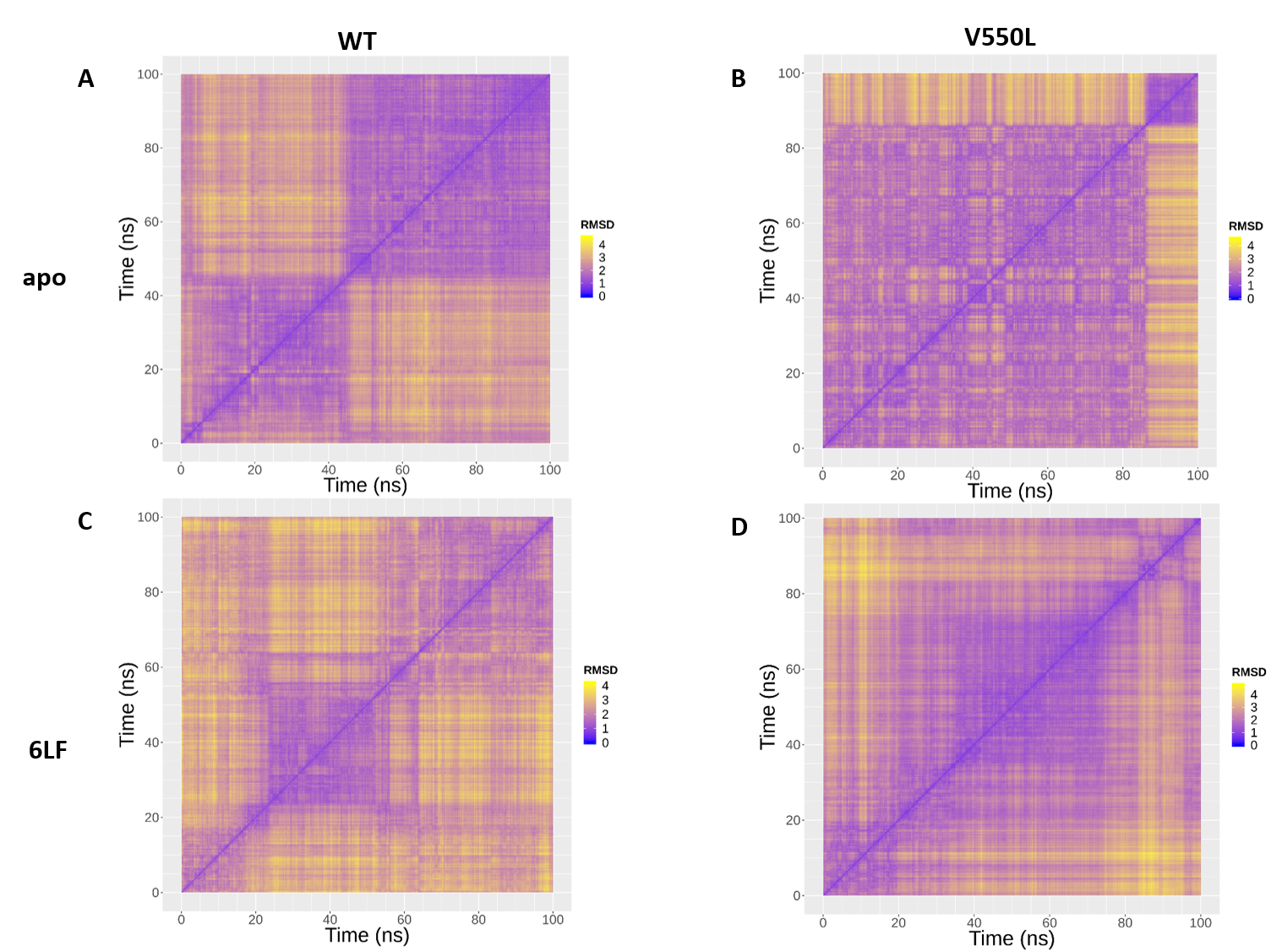


**Supplementary Figure 1. The pairwise RMSD results indicate that the 100 ns of the simulation is not enough.** The systems highly fluctuated during the first 100 ns to reach their stable conformation.


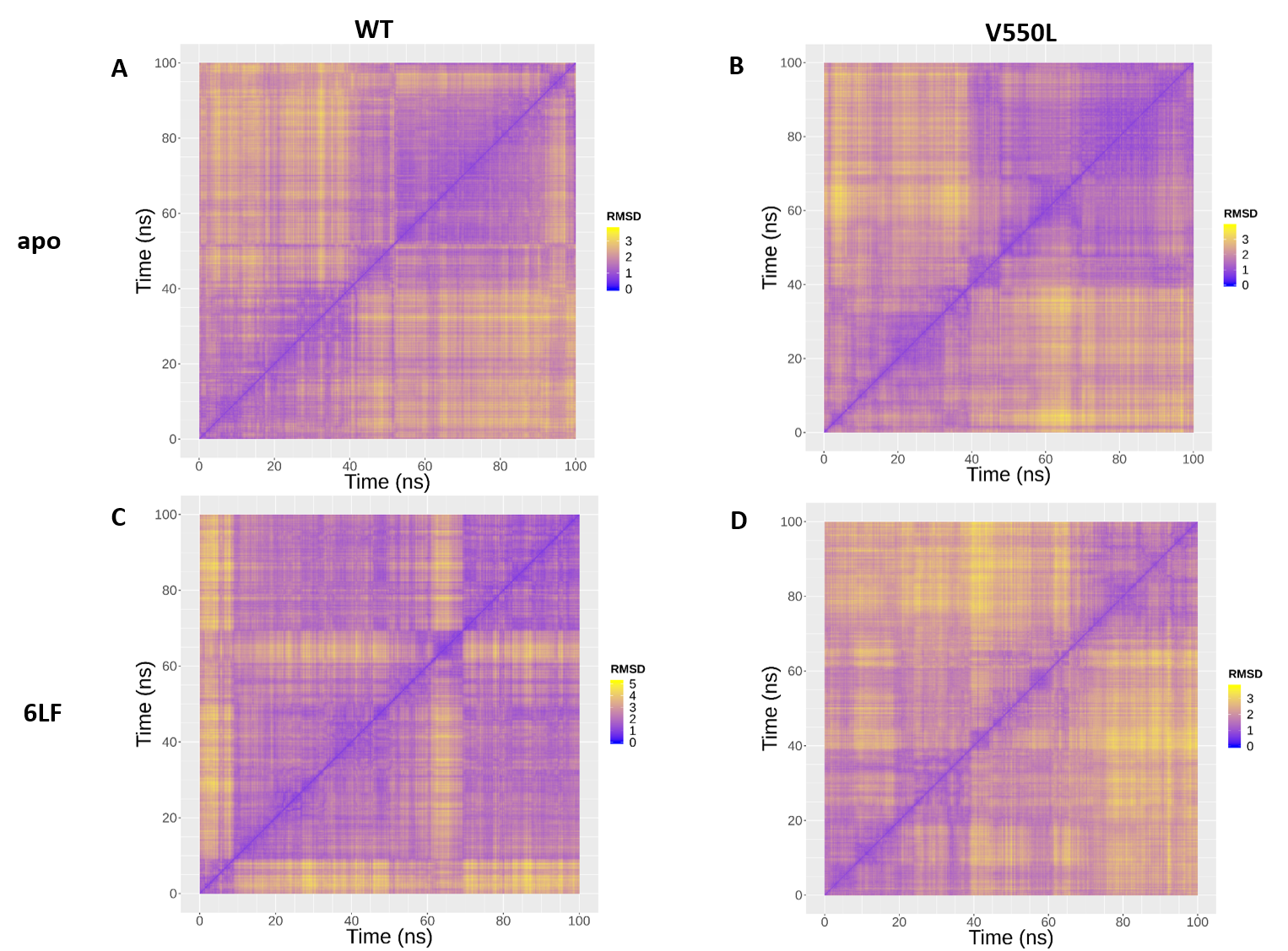


**Supplementary Figure 2.** **The pairwise RMSD analysis for the second 100 ns shows a relatively stable conformation of all systems.**

**Supplementary Tables:**

**Supplementary Table1. Different steps of each simulation and the selected one for comparison of four systems. The selected one is underlined.**

| **Simulated complexes** | apo_WT_FGFR4 | apo_V550L_FGFR4 |
| --- | --- | --- |
| **Conformational steps based on pairwise RMASD** | 0-50  50-90  90-100 | 0-50  50-100 |
| **Simulated complexes** | 6LF-WT_FGFR4 RMSD | 6LF-V550L_FGFR4 RMSD |
| **Conformational steps based on pairwise RMASD** | 0-10  10-70  70-100 | 0-40  40-70  70-100 |

**Supplementary Table2.** The most important residues in 6LF binding in both WT and V550L complexes are highlighted in yellow. The most important differences in binding patterns all are also highlighted in blue.

|  |  | TOTAL WT | TOTAL V550L |
| --- | --- | --- | --- |
| Resid 1 | Resid 2 |  |  |
| 6LF 298 | LEU 1 | -0.000330335 | 0.000209395 |
| 6LF 298 | PRO 2 | -0.000267866 | -1.25E-05 |
| 6LF 298 | LEU 3 | -0.001610695 | -6.50E-06 |
| 6LF 298 | ASP 4 | -0.000792104 | -0.000346327 |
| 6LF 298 | PRO 5 | -4.55E-05 | -8.00E-06 |
| 6LF 298 | LEU 6 | -0.000744128 | -0.000468766 |
| 6LF 298 | TRP 7 | -0.0027991 | -0.002342829 |
| 6LF 298 | GLU 8 | -0.001784108 | -0.001494753 |
| 6LF 298 | PHE 9 | -0.001995002 | -0.001886057 |
| 6LF 298 | PRO 10 | -0.00102049 | -0.000868566 |
| 6LF 298 | ARG 11 | -0.000798101 | -0.001304348 |
| 6LF 298 | ASP 12 | -0.000781109 | 0.000176412 |
| 6LF 298 | ARG 13 | -0.000427286 | -0.00134083 |
| 6LF 298 | LEU 14 | -0.00673913 | -0.007831584 |
| 6LF 298 | VAL 15 | -0.004125437 | -0.003471764 |
| 6LF 298 | LEU 16 | -0.012943528 | -0.012245877 |
| 6LF 298 | GLY 17 | -0.019028986 | -0.012705647 |
| 6LF 298 | LYS 18 | -0.549484033 | -0.144446028 |
| 6LF 298 | PRO 19 | -0.219288035 | -0.222146368 |
| 6LF 298 | LEU 20 | -5.658394168 | -5.000377043 |
| 6LF 298 | GLY 21 | -1.130749756 | -1.01407437 |
| 6LF 298 | GLU 22 | -3.325537129 | -0.487666534 |
| 6LF 298 | GLY 23 | -0.074941322 | -0.147400449 |
| 6LF 298 | CYS 24 | -0.017715142 | -0.014383976 |
| 6LF 298 | PHE 25 | -0.013763118 | -0.011004498 |
| 6LF 298 | GLY 26 | -0.037151435 | -0.042154054 |
| 6LF 298 | GLN 27 | -0.133228176 | -0.183644094 |
| 6LF 298 | VAL 28 | -2.744238712 | -3.342355134 |
| 6LF 298 | VAL 29 | -0.236376479 | -0.149264561 |
| 6LF 298 | ARG 30 | -0.641327595 | -0.363233859 |
| 6LF 298 | ALA 31 | -0.000317841 | 0.002766617 |
| 6LF 298 | GLU 32 | -0.004333333 | 0.001713143 |
| 6LF 298 | ALA 33 | -0.001575212 | -0.000936032 |
| 6LF 298 | PHE 34 | -0.001088956 | -0.001004998 |
| 6LF 298 | GLY 35 | -0.000524238 | -0.00045927 |
| 6LF 298 | MET 36 | -0.004231884 | -0.004621689 |
| 6LF 298 | ASP 37 | -0.004294853 | -0.002162419 |
| 6LF 298 | PRO 38 | -0.000655172 | -0.000524738 |
| 6LF 298 | ALA 39 | -0.000374813 | -0.000563218 |
| 6LF 298 | ARG 40 | -0.00123938 | -0.00410045 |
| 6LF 298 | PRO 41 | -0.000247376 | -0.000128936 |
| 6LF 298 | ASP 42 | -0.000470765 | 0.000541229 |
| 6LF 298 | GLN 43 | -0.001093453 | -0.001035482 |
| 6LF 298 | ALA 44 | -0.0014003 | -0.001521739 |
| 6LF 298 | SER 45 | -0.008974013 | -0.00898051 |
| 6LF 298 | THR 46 | -0.079152692 | -0.101681768 |
| 6LF 298 | VAL 47 | -0.139960123 | -0.210342963 |
| 6LF 298 | ALA 48 | -1.160707998 | -1.306689537 |
| 6LF 298 | VAL 49 | -0.140949824 | -0.152102951 |
| 6LF 298 | LYS 50 | -1.26122782 | -0.52069725 |
| 6LF 298 | MET 51 | -0.018471264 | -0.008562719 |
| 6LF 298 | LEU 52 | -0.008636182 | -0.006773613 |
| 6LF 298 | LYS 53 | -0.009044478 | -0.004101949 |
| 6LF 298 | ASP 54 | -0.001708146 | -0.002537731 |
| 6LF 298 | ASN 55 | -0.001635682 | -0.001389805 |
| 6LF 298 | ALA 56 | -0.001398301 | -0.000944528 |
| 6LF 298 | SER 57 | -0.00104048 | -0.000850075 |
| 6LF 298 | ASP 58 | -0.001023488 | -0.000785107 |
| 6LF 298 | LYS 59 | -0.00176012 | -9.65E-05 |
| 6LF 298 | ASP 60 | -0.00444078 | -0.004272364 |
| 6LF 298 | LEU 61 | -0.002457771 | -0.001775612 |
| 6LF 298 | ALA 62 | -0.001343828 | -0.001008996 |
| 6LF 298 | ASP 63 | -0.008633183 | -0.004953023 |
| 6LF 298 | LEU 64 | -0.023270865 | -0.01767916 |
| 6LF 298 | VAL 65 | -0.001977511 | -0.001072964 |
| 6LF 298 | SER 66 | -0.00416042 | -0.001530735 |
| 6LF 298 | GLU 67 | -0.064948648 | -0.023295852 |
| 6LF 298 | MET 68 | -0.004275862 | -0.006097951 |
| 6LF 298 | GLU 69 | -0.002233883 | -0.000969015 |
| 6LF 298 | VAL 70 | -0.011178411 | -0.005778111 |
| 6LF 298 | MET 71 | -0.031041192 | -0.026408068 |
| 6LF 298 | LYS 72 | -0.000149925 | 0.000948526 |
| 6LF 298 | LEU 73 | -0.001591704 | -0.000889555 |
| 6LF 298 | ILE 74 | -0.007991004 | -0.005787606 |
| 6LF 298 | GLY 75 | -0.001547726 | -0.001377811 |
| 6LF 298 | ARG 76 | -0.002308346 | -0.003162419 |
| 6LF 298 | HIE 77 | -0.004154423 | -0.004055472 |
| 6LF 298 | LYS 78 | -0.013427286 | -0.015864568 |
| 6LF 298 | ASN 79 | -0.029650675 | -0.030752624 |
| 6LF 298 | ILE 80 | -0.099144428 | -0.091572214 |
| 6LF 298 | ILE 81 | -1.759748339 | -1.513517478 |
| 6LF 298 | ASN 82 | -0.05585907 | -0.052994697 |
| 6LF 298 | LEU 83 | -0.022630685 | -0.027603198 |
| 6LF 298 | LEU 84 | -0.020791104 | -0.023572714 |
| 6LF 298 | GLY 85 | -0.004126437 | -0.003761119 |
| 6LF 298 | VAL 86 | -0.004336332 | -0.005235382 |
| 6LF 298 | CYS 87 | -0.001311844 | -0.000787106 |
| 6LF 298 | THR 88 | -0.000854573 | -0.000848576 |
| 6LF 298 | GLN 89 | -0.000286357 | 5.00E-06 |
| 6LF 298 | GLU 90 | -0.000512744 | -0.000101949 |
| 6LF 298 | GLY 91 | -2.40E-05 | -3.15E-05 |
| 6LF 298 | PRO 92 | -0.001194903 | -0.001114443 |
| 6LF 298 | LEU 93 | -0.007383308 | -0.008606697 |
| 6LF 298 | TYR 94 | -0.01708096 | -0.021646177 |
| 6LF 298 | VAL 95 | -0.044838581 | -0.052995002 |
| 6LF 298 | ILE 96 | -0.047653673 | -0.075793103 |
| 6LF 298 | VAL 97 | -0.640258594 | -0.998539386 |
| 6LF 298 | GLU 98 | -3.505158461 | -3.391640498 |
| 6LF 298 | CYS 99 | -2.431162977 | -2.52154584 |
| 6LF 298 | ALA 100 | -3.474687984 | -3.452432936 |
| 6LF 298 | ALA 101 | -0.731150916 | -0.803169478 |
| 6LF 298 | LYS 102 | -0.753444642 | -1.129937003 |
| 6LF 298 | GLY 103 | -2.701286224 | -3.338808818 |
| 6LF 298 | ASN 104 | -2.22731386 | -5.571869609 |
| 6LF 298 | LEU 105 | -0.010019814 | -0.074582026 |
| 6LF 298 | ARG 106 | -0.085875167 | -0.121189784 |
| 6LF 298 | GLU 107 | -0.186467166 | -0.457769558 |
| 6LF 298 | PHE 108 | -0.03210271 | -0.076460137 |
| 6LF 298 | LEU 109 | -0.006467266 | -0.015785107 |
| 6LF 298 | ARG 110 | -0.010129935 | -0.009682659 |
| 6LF 298 | ALA 111 | -0.004988006 | -0.007697651 |
| 6LF 298 | ARG 112 | -0.003666667 | -0.01442029 |
| 6LF 298 | ARG 113 | -0.000671164 | -0.001863068 |
| 6LF 298 | PRO 114 | -8.10E-05 | -0.000589705 |
| 6LF 298 | PRO 115 | 7.50E-06 | -9.30E-05 |
| 6LF 298 | GLY 116 | 4.50E-06 | 2.85E-05 |
| 6LF 298 | PRO 117 | 2.00E-06 | 2.65E-05 |
| 6LF 298 | ASP 118 | -0.000109945 | -1.10E-05 |
| 6LF 298 | LEU 119 | 5.50E-06 | 3.50E-05 |
| 6LF 298 | SER 120 | -2.00E-06 | -1.00E-05 |
| 6LF 298 | PRO 121 | -3.00E-06 | -2.20E-05 |
| 6LF 298 | ASP 122 | 8.00E-06 | 0.000192404 |
| 6LF 298 | GLY 123 | 5.00E-07 | 7.00E-06 |
| 6LF 298 | PRO 124 | 7.00E-06 | 1.80E-05 |
| 6LF 298 | ARG 125 | -4.45E-05 | -0.000205897 |
| 6LF 298 | SER 126 | 8.50E-06 | 2.40E-05 |
| 6LF 298 | SER 127 | 4.50E-06 | -3.00E-06 |
| 6LF 298 | GLU 128 | 0.000134433 | 0.00076062 |
| 6LF 298 | GLY 129 | -9.00E-06 | 2.10E-05 |
| 6LF 298 | PRO 130 | -0.00053973 | -0.000909045 |
| 6LF 298 | LEU 131 | -0.001171414 | -0.001971514 |
| 6LF 298 | SER 132 | 8.40E-05 | 8.55E-05 |
| 6LF 298 | PHE 133 | 4.55E-05 | 8.15E-05 |
| 6LF 298 | PRO 134 | 6.45E-05 | 6.80E-05 |
| 6LF 298 | VAL 135 | -0.001033983 | -0.001267366 |
| 6LF 298 | LEU 136 | -0.00098051 | -0.001184408 |
| 6LF 298 | VAL 137 | -0.000223888 | -0.000993503 |
| 6LF 298 | SER 138 | -0.001236382 | -0.001602699 |
| 6LF 298 | CYS 139 | -0.003216392 | -0.004858571 |
| 6LF 298 | ALA 140 | -0.000932534 | -0.000833583 |
| 6LF 298 | TYR 141 | -0.001295852 | -0.001462769 |
| 6LF 298 | GLN 142 | -0.006111944 | -0.006644178 |
| 6LF 298 | VAL 143 | -0.00974063 | -0.010118441 |
| 6LF 298 | ALA 144 | -0.000557221 | -0.000548726 |
| 6LF 298 | ARG 145 | -0.002755622 | -0.002477261 |
| 6LF 298 | GLY 146 | -0.004408796 | -0.003012994 |
| 6LF 298 | MET 147 | -0.00235982 | -0.001436282 |
| 6LF 298 | GLN 148 | -0.000774613 | -0.000870565 |
| 6LF 298 | TYR 149 | -0.003358821 | -0.002368816 |
| 6LF 298 | LEU 150 | -0.01097901 | -0.006584208 |
| 6LF 298 | GLU 151 | -0.000152924 | -0.000918541 |
| 6LF 298 | SER 152 | -0.000357321 | -1.45E-05 |
| 6LF 298 | ARG 153 | -0.001394803 | -0.000578211 |
| 6LF 298 | LYS 154 | -0.001502249 | -0.000481259 |
| 6LF 298 | CYS 155 | -0.00383908 | -0.001507246 |
| 6LF 298 | ILE 156 | -0.010027986 | -0.0025992 |
| 6LF 298 | HIE 157 | -0.035252874 | -0.019763753 |
| 6LF 298 | ARG 158 | -0.017485257 | -0.002597201 |
| 6LF 298 | ASP 159 | -0.037021694 | -0.012607196 |
| 6LF 298 | LEU 160 | -0.019388306 | -0.013126937 |
| 6LF 298 | ALA 161 | -0.023150925 | -0.035189905 |
| 6LF 298 | ALA 162 | -0.05014043 | -0.060114714 |
| 6LF 298 | ARG 163 | -0.945958533 | -0.73796081 |
| 6LF 298 | ASN 164 | -0.249606638 | -0.29404082 |
| 6LF 298 | VAL 165 | -0.215850123 | -0.42390791 |
| 6LF 298 | LEU 166 | -3.413636627 | -5.45153148 |
| 6LF 298 | VAL 167 | -0.131921779 | -0.15972526 |
| 6LF 298 | THR 168 | -0.063762619 | -0.084228386 |
| 6LF 298 | GLU 169 | 0.005793302 | 0.022335332 |
| 6LF 298 | ASP 170 | -0.002457271 | 0.002755622 |
| 6LF 298 | ASN 171 | -0.011887056 | -0.015858571 |
| 6LF 298 | VAL 172 | -0.029021989 | -0.036161919 |
| 6LF 298 | MET 173 | -0.05289905 | -0.060903048 |
| 6LF 298 | LYS 174 | -0.170209833 | -0.163361187 |
| 6LF 298 | ILE 175 | -0.145030985 | -0.132464495 |
| 6LF 298 | ALA 176 | -0.963706202 | -1.110269956 |
| 6LF 298 | ASP 177 | -1.353975387 | -0.983605301 |
| 6LF 298 | PHE 178 | -3.92695874 | -3.572725046 |
| 6LF 298 | GLY 179 | -0.067854027 | -0.0327233 |
| 6LF 298 | LEU 180 | -0.01421939 | -0.020485757 |
| 6LF 298 | ALA 181 | -0.051648804 | -0.001268366 |
| 6LF 298 | ARG 182 | -1.496907029 | -0.019557221 |
| 6LF 298 | GLY 183 | -0.00221939 | -0.000988506 |
| 6LF 298 | VAL 184 | -0.002647676 | -0.001645677 |
| 6LF 298 | HIE 185 | -0.001963018 | -0.000588206 |
| 6LF 298 | HIE 186 | -0.004532734 | -0.000895552 |
| 6LF 298 | ILE 187 | -0.00842029 | -0.001044978 |
| 6LF 298 | ASP 188 | -0.00026037 | -0.000831084 |
| 6LF 298 | TYR 189 | -0.001265367 | -0.000342829 |
| 6LF 298 | TYR 190 | -0.000930035 | -0.000184908 |
| 6LF 298 | LYS 191 | -0.002557221 | 0.000216892 |
| 6LF 298 | LYS 192 | -0.004594703 | -0.000262369 |
| 6LF 298 | THR 193 | -0.025503577 | -0.00806097 |
| 6LF 298 | SER 194 | -0.131239296 | -0.029981523 |
| 6LF 298 | ASN 195 | -0.392204798 | -0.094360473 |
| 6LF 298 | GLY 196 | -0.00637931 | -0.005327836 |
| 6LF 298 | ARG 197 | -0.029848076 | -0.01924038 |
| 6LF 298 | LEU 198 | -0.00281959 | -0.001809095 |
| 6LF 298 | PRO 199 | -0.003261369 | -0.00081959 |
| 6LF 298 | VAL 200 | -0.000908546 | -0.000435282 |
| 6LF 298 | LYS 201 | -0.00230035 | -0.000110445 |
| 6LF 298 | TRP 202 | -0.005766617 | -0.004075462 |
| 6LF 298 | MET 203 | -0.001487256 | -0.000962019 |
| 6LF 298 | ALA 204 | -4.15E-05 | -3.40E-05 |
| 6LF 298 | PRO 205 | 1.90E-05 | -8.00E-06 |
| 6LF 298 | GLU 206 | 0.000723138 | -7.35E-05 |
| 6LF 298 | ALA 207 | -0.000144428 | -1.05E-05 |
| 6LF 298 | LEU 208 | -0.000167916 | -1.55E-05 |
| 6LF 298 | PHE 209 | -4.50E-06 | -4.50E-06 |
| 6LF 298 | ASP 210 | 0.000362819 | -9.35E-05 |
| 6LF 298 | ARG 211 | -0.001192404 | 0.000332334 |
| 6LF 298 | VAL 212 | 1.65E-05 | 3.00E-06 |
| 6LF 298 | TYR 213 | -0.002106947 | -0.000557721 |
| 6LF 298 | THR 214 | -0.00029935 | 4.50E-06 |
| 6LF 298 | HIE 215 | -0.000690155 | 8.67E-22 |
| 6LF 298 | GLN 216 | -0.000127936 | 1.40E-05 |
| 6LF 298 | SER 217 | -0.000806097 | -7.00E-06 |
| 6LF 298 | ASP 218 | 3.00E-06 | -0.001072464 |
| 6LF 298 | VAL 219 | -0.000738631 | -2.90E-05 |
| 6LF 298 | TRP 220 | -0.001209895 | -0.000882059 |
| 6LF 298 | SER 221 | -0.002363818 | -0.001507746 |
| 6LF 298 | PHE 222 | -0.001813093 | -0.001646177 |
| 6LF 298 | GLY 223 | -3.10E-05 | -2.05E-05 |
| 6LF 298 | ILE 224 | -0.002272864 | -0.002329835 |
| 6LF 298 | LEU 225 | -0.008464268 | -0.009866567 |
| 6LF 298 | LEU 226 | -0.000725137 | -0.00055922 |
| 6LF 298 | TRP 227 | -0.001111444 | -0.001294353 |
| 6LF 298 | GLU 228 | -0.001734633 | -0.005923038 |
| 6LF 298 | ILE 229 | -0.002668166 | -0.003824588 |
| 6LF 298 | PHE 230 | -0.000882059 | -0.000638681 |
| 6LF 298 | THR 231 | -0.000993503 | -0.001155922 |
| 6LF 298 | LEU 232 | -0.002111944 | -0.00536032 |
| 6LF 298 | GLY 233 | -0.001370815 | -0.00195902 |
| 6LF 298 | GLY 234 | -0.000787606 | -0.000776612 |
| 6LF 298 | SER 235 | -0.001133933 | -0.001029985 |
| 6LF 298 | PRO 236 | -0.000942029 | -0.00072014 |
| 6LF 298 | TYR 237 | -0.000547226 | -0.000331834 |
| 6LF 298 | PRO 238 | -2.25E-05 | 3.85E-05 |
| 6LF 298 | GLY 239 | -2.10E-05 | 1.90E-05 |
| 6LF 298 | ILE 240 | 2.55E-05 | -3.45E-05 |
| 6LF 298 | PRO 241 | 1.00E-05 | 3.40E-05 |
| 6LF 298 | VAL 242 | -9.00E-06 | 4.80E-05 |
| 6LF 298 | GLU 243 | 0.000274863 | -0.00044028 |
| 6LF 298 | GLU 244 | 0.000216892 | -0.000212394 |
| 6LF 298 | LEU 245 | -1.50E-05 | 3.50E-05 |
| 6LF 298 | PHE 246 | 0 | 2.05E-05 |
| 6LF 298 | SER 247 | -4.00E-06 | 1.70E-05 |
| 6LF 298 | LEU 248 | -1.30E-05 | 2.30E-05 |
| 6LF 298 | LEU 249 | -1.55E-05 | 1.80E-05 |
| 6LF 298 | ARG 250 | -0.000269365 | 0.000132434 |
| 6LF 298 | GLU 251 | 0.00018091 | -6.25E-05 |
| 6LF 298 | GLY 252 | -1.40E-05 | 9.50E-06 |
| 6LF 298 | HIE 253 | 1.20E-05 | 1.60E-05 |
| 6LF 298 | ARG 254 | -0.000353823 | -4.50E-06 |
| 6LF 298 | MET 255 | -1.80E-05 | -6.00E-06 |
| 6LF 298 | ASP 256 | 0.000342829 | 8.00E-06 |
| 6LF 298 | ARG 257 | -0.000278361 | -0.000194403 |
| 6LF 298 | PRO 258 | -5.10E-05 | -2.50E-06 |
| 6LF 298 | PRO 259 | 4.25E-05 | 5.10E-05 |
| 6LF 298 | HIE 260 | 1.45E-05 | 4.05E-05 |
| 6LF 298 | CYS 261 | -1.45E-05 | -2.45E-05 |
| 6LF 298 | PRO 262 | 3.35E-05 | 1.10E-05 |
| 6LF 298 | PRO 263 | 2.15E-05 | 6.00E-06 |
| 6LF 298 | GLU 264 | 0.000254873 | 0.000188906 |
| 6LF 298 | LEU 265 | 1.35E-05 | -1.45E-05 |
| 6LF 298 | TYR 266 | 4.50E-06 | 5.00E-07 |
| 6LF 298 | GLY 267 | 1.15E-05 | -1.15E-05 |
| 6LF 298 | LEU 268 | 5.50E-06 | -2.80E-05 |
| 6LF 298 | MET 269 | 1.20E-05 | -3.45E-05 |
| 6LF 298 | ARG 270 | -0.000277361 | -7.20E-05 |
| 6LF 298 | GLU 271 | 0.000331834 | 0.000102949 |
| 6LF 298 | CYS 272 | 1.65E-05 | -5.00E-06 |
| 6LF 298 | TRP 273 | -0.000250875 | -1.85E-05 |
| 6LF 298 | HIE 274 | 3.85E-05 | -1.10E-05 |
| 6LF 298 | ALA 275 | 3.50E-05 | 0 |
| 6LF 298 | ALA 276 | 1.30E-05 | 9.50E-06 |
| 6LF 298 | PRO 277 | 6.50E-06 | 1.05E-05 |
| 6LF 298 | SER 278 | 0 | 4.50E-06 |
| 6LF 298 | GLN 279 | -4.50E-06 | 5.50E-06 |
| 6LF 298 | ARG 280 | -0.000709645 | -2.70E-05 |
| 6LF 298 | PRO 281 | 4.40E-05 | -8.00E-06 |
| 6LF 298 | THR 282 | -1.10E-05 | 2.60E-05 |
| 6LF 298 | PHE 283 | -0.001132434 | -0.000770115 |
| 6LF 298 | LYS 284 | -0.000267366 | -0.000109945 |
| 6LF 298 | GLN 285 | -4.00E-06 | 1.00E-05 |
| 6LF 298 | LEU 286 | -0.000168416 | -1.00E-06 |
| 6LF 298 | VAL 287 | -3.05E-05 | 2.55E-05 |
| 6LF 298 | GLU 288 | 0.000274363 | 0.00030035 |
| 6LF 298 | ALA 289 | -3.25E-05 | -3.50E-06 |
| 6LF 298 | LEU 290 | -6.80E-05 | -2.90E-05 |
| 6LF 298 | ASP 291 | 0.000321339 | 0.000555222 |
| 6LF 298 | LYS 292 | -0.000212394 | -0.000242379 |
| 6LF 298 | VAL 293 | -3.00E-05 | -1.60E-05 |
| 6LF 298 | LEU 294 | -2.35E-05 | -1.25E-05 |
| 6LF 298 | LEU 295 | -3.50E-06 | -6.00E-06 |
| 6LF 298 | ALA 296 | -9.00E-06 | -7.00E-06 |
| 6LF 298 | VAL 297 | 0.000144928 | 0.000302849 |
| 6LF 298 | 6LF 298 | 30.48937111 | 29.90979281 |
